# Extensive deep vein thrombosis in a young parturient with a brief use of oral contraceptive pills

**DOI:** 10.4103/0019-5049.71031

**Published:** 2010

**Authors:** Jayashree C Patki, C Naresh K Reddy

**Affiliations:** Department of Anesthesiology, Krishna Institute of Medical Sciences, Secunderabad - 500 003, India

Sir,

Because of misleading publicity by the media and over-the-counter availability of oral contraceptive (OC) pills, its use is increased all over the world, which can result in fatal complications like deep vein thrombosis (DVT). DVT occurs in 2–3/1000 patients who give history of use of OC pills for various reasons. As pregnant patients are from younger age group, risk of DVT often gets ignored during the pre-anaesthetic checkup. Once diagnosed, these patients are managed by conventional anticoagulation which may lead to change in anaesthesia technique and becomes a challenge for the anaesthetist during emergencies.

A 28-year-old female, weighing 48 kg, with a diagnosis of 3rd gravida with extensive DVT, was scheduled for elective Lower Segment Caesarean Section (LSCS). She had a history of use of OC pills on and off for irregular periods, with no other co-morbidities. Laboratory investigations were normal except raised activated partial thromboplastin time (APTT). Lupus anticoagulants were on higher side. X-ray chest, electrocardiogram (ECG) and echocardiography were normal. Venous Doppler showed deep venous thrombosis of external iliac, femoral, poplitial and anterior tibial veins. There was presence of extensive thrombus in great saphanous vein. She was on low molecular weight heparin (LMWH) which was stopped a day before surgery. Also, 2 units each of compatible blood and fresh frozen plasma (FFP) were reserved for emergency use.

General anaesthesia was planned. After taking informed consent, she was shifted to operation theatre for caesarean section. General anaesthesia was induced with thiopentone sodium 250 mg and succinyl choline 100 mg intravenously. Trachea was intubated with cuffed endotracheal tube of size 7.0 mm. Anaesthesia was maintained with O_2_:N_2_O (40:60), and vecuronium bromide (4 mg). After delivery of baby, Inj. Fentanyl 100 mcg, Oxytocin 10 U and Inj. Midazolam 1 mg IV were administered. She received approximately 1000 ml of crystalloid intraoperatively. Total blood loss was approximately 500 ml. At the end of surgery, reversal of neuromuscular blockade was achieved with Inj. Neostigmine 2.5 mg and Inj. Glycopyrolate 0.4 mg. After extubation, she was shifted to PICU and closely monitored. LMWH was withheld till the next day, considering the risk of postoperative bleeding. Her subsequent course in the hospital was uneventful and she was referred to the cardiologist for further management.

Every patient with even a brief history of use of OC pills should be evaluated thoroughly. There is an increased risk of VTE due to OC use in women with elevated factor VIII, and raised level of the coagulation factor and OC use seem to have a synergistic effect.[1] DVT may give rise to pulmonary embolism, which has a maternal mortality of 1:100,000.[2] In women with clinically suspected DVT, the diagnosis should be confirmed by investigation. Initial investigation is by Doppler ultrasonography. If inconclusive, serial testing by ultrasonography or contrast venography (with abdominal shielding) can be done. D-dimmer values should be checked to confirm the diagnosis. Blood should be checked for APTT; complete blood picture, thrombophilia screen, coagulation studies, urea and electrolytes and liver function tests need to be taken to exclude renal or hepatic dysfunction.[3] A prolonged APTT should raise the suspicion of a lupus anticoagulant being present.

Following confirmation of diagnosis of DVT, the patient should be informed about the diagnosis and the complications. Such patients might be on anticoagulants which interfere with the anaesthesia techniques and pose a challenge to the anaesthetist.

Problems encountered by the anaesthesiologist in the management of symptomatic patients include:

1. risk of intraoperative bleeding and risk of postpartum haemorrhage because of use of LMWH and
2. risk of pulmonary embolism if anticoagulants are stopped.

Our anaesthetic goals should be as follows:

1. In view of the presence of extensive DVT, to continue LMWH till 12 hours before surgery.
2. Reserve adequate quantity of compatible blood and FFP. If APTT is still prolonged, then FFP should be administered to normalise the APTT.
3. Ensure that anti embolic stockings are in place before caesarean section.
4. Keep the patient well hydrated.
5. Opt for general anaesthesia instead of spinal/epidural.

Regional anaesthesia (epidural or spinal) is to be avoided during anticoagulation treatment because of risk of spinal haematoma.

Recommence anticoagulation treatment at the same dose regimen given antenatally in the postpartum period. Neither oral anticoagulants nor heparin appears in breast milk to any significant degree and breast feeding is not contraindicated. LMWH is the agent of choice as it binds less to platelet factor 4, substantially reducing the risk of heparin-induced thrombocytopaenia.[4] Patients with a history of previous VTE and acquired thrombophilia (antiphospholipid syndrome) have up to 70% chances of recurrent thrombosis, even higher in pregnancy. Some of these women will be on long-term oral anticoagulants outside pregnancy. These women need antenatal, intrapartum and postnatal prophylactic thromboprophylaxis.

To conclude, proper evaluation of patients with history of even brief use of OC pills is required to decide on the anaesthetic technique and proper and adequate perioperative care to prevent maternal morbidity and mortality.
